# Frictional adhesion of geckos predicts maximum running performance in nature

**DOI:** 10.1242/jeb.247906

**Published:** 2025-01-09

**Authors:** Timothy E. Higham

**Affiliations:** Department of Evolution, Ecology, and Organismal Biology, University of California, Riverside, CA 92521, USA

**Keywords:** Namibia, *Rhoptropus*, Pachydactylus, Rock, Roughness, Acceleration, Speed

## Abstract

Despite the myriad studies examining the diversity and mechanisms of gecko adhesion in the lab, we have a poor understanding of how this translates to locomotion in nature. It has long been assumed that greater adhesive strength should translate to superior performance in nature. Using 13 individuals of Bradfield's Namib day gecko (*Rhoptropus bradfieldi*) in Namibia, I tested the hypothesis that maximum running performance in nature (speed and acceleration) is driven by maximum frictional adhesive strength. Specifically, those individuals with greater frictional adhesion should escape with faster speed and acceleration because of increased contact with the surface from which to apply propulsive forces. I tested this prediction by quantifying laboratory adhesive performance and then releasing the geckos into the field while simultaneously recording the escape using high-speed videography. Additional measurements included how this species modulates maximum running speed (stride length and/or stride frequency) and how temperature influences field performance. I found that maximum acceleration was significantly correlated with maximum frictional adhesive strength, whereas maximum sprinting speed was only correlated with increases in stride frequency (not stride length) and temperature. Thus, different measures of performance (acceleration and speed) are limited by very different variables. Acceleration is key for rapidly escaping predation and, given their correlation, maximum frictional adhesion likely plays a key role in fitness.

## Teaser

**Summary:** Maximum gecko acceleration in nature is predicted by maximum adhesive force, whereas maximum running speed is predicted only by temperature and not adhesion, suggesting that adhesion is an ecologically relevant trait in geckos.

## INTRODUCTION

The ability to execute an ecologically relevant task (i.e. organismal performance) depends on the integration of multiple functional traits (Irschick and Higham, 2016). This is central to ecomechanics (also termed mechanical ecology), which examines the mechanisms underlying the interactions of organisms with their biotic and abiotic environment (Bauer et al., 2020; Higham et al., 2021a; Ferry and Higham, 2022). To understand how animals perform in nature, laboratory studies often serve as a proxy. Comparatively fewer studies have measured and compared laboratory and field performance/behavior (Irschick, 2003; Higham, 2019). In some cases, maximum performance, measured in the laboratory, does not directly predict performance in nature. For example, Irschick and Losos (1998) examined maximum sprinting and jumping performance of eight species of *Anolis* lizards in the laboratory and found that sprint performance in nature was 90%, 32% and 71% of maximum when escaping, during undisturbed activity and during feeding, respectively. For fast lizards that live in open terrestrial habitats, laboratory measures of performance can underestimate those in the field. For example, Jayne and Ellis (1998) found that laboratory sprint performance of the Mojave fringe-toed lizard (*Uma scoparia*) was between 71 and 77% of estimated field sprint performance (using stride length as a proxy for speed). Both of these examples highlight the potential disconnect between laboratory and field performance.

Micro- and macrohabitat use differences could also influence performance and thus fitness, in nature. This is probably the clearest for arboreal anoles from the Greater Antilles (Losos, 1990; Irschick et al., 1997). In this case, limb length is strongly correlated with microhabitat use, and is also associated with differences in performance, such as running (Irschick et al., 2005). In a large study of 19 species of lizard from different microhabitats (from rocks to leaf litter), rock-dwelling lizards had longer limbs and sprinted faster (Goodman et al., 2008). The inclination of the substrate is another well-known factor that impacts running performance (Irschick and Jayne, 1998; Pinch and Claussen, 2003; Warner and Shine, 2006; Collins and Higham, 2017), with steeper slopes often resulting in decreased running speed. Sleeper slopes also require an animal to cling to the substrate to avoid sliding or toppling backwards (Cartmill, 1985). Mechanisms for clinging in vertebrates include claws, adhesive systems, prehensile hands or feet for grasping, or modifying kinematics to increase the normal force. Ultimately, the interplay between the habitat, morphological/behavioral specializations of the animal and physiology will determine locomotor performance.

Intraspecific variation in functional traits related to the locomotor system of lizards are often used to predict maximum sprinting performance. Several studies have found that individuals/species/populations of lizards with longer legs sprint faster on broad surfaces (trackways and treadmills) in the laboratory (Sinervo and Losos, 1991; Garland and Losos, 1994; Macrini and Irschick, 1998; Bonine and Garland, 1999; Goodman et al., 2008). However, variation in limb-related functional traits do not always predict intraspecific variation in performance. Among hatchling *Amphibolurus muricatus* lizards, the links between morphology and performance were weak (Warner and Shine, 2006). In addition to morphological variation driving differences in performance, kinematics of individual joints may also be a major contributor. In a recent study of a Namibian day gecko, *Rhoptropus afer*, path analyses were used to determine the relative contribution of kinematic traits to sprinting performance (Collins and Higham, 2017). As predicted, the movements of distal joints and segments, predominantly ankle extension, were the primary drivers of variation in sprint speed among the 33 individuals used in the study. For a climbing vertebrate, one might expect traits related to clinging performance to be good predictors of performance.

While the above-mentioned studies are extremely important, few determine which traits are responsible for performance in nature (but see Irschick and Losos, 1998, Braña, 2003, Husak, 2006, Husak and Fox, 2006, and Stiller and McBrayer, 2013 for examples). For a climbing animal, this could include traits related to clinging ability, such as toepad area (Winchell et al., 2018). Although many organisms use adhesion to remain stationary on a surface (Wainwright et al., 2013; Beckert et al., 2015), others use adhesion to remain attached while climbing (Cartmill, 1985; Blob et al., 2008; Labonte and Federle, 2015; Federle and Labonte, 2019). This is termed adhesive locomotion and requires significant coordination between the attachment system and the movement of the appendages. A key question that remains poorly understood is what determines intraspecific variation in climbing ability (in nature) among organisms that rely on adhesion?

Geckos are noteworthy for their ability to temporarily and reversibly adhere to vertical or even inverted smooth surfaces (Niewiarowski et al., 2016; Russell et al., 2019; Higham and Russell, 2025). To do this, they use integumentary outgrowths on the ventral side of their digits, termed setae. Setae are directional and controlled by a series of hierarchical anatomical components, including digital tendons (Russell, 1976), derived ankle structure (Higham et al., 2021b), vascular sinuses (Russell, 1981) and complex musculature (Russell, 1975). Adhesion is ultimately dependent upon contact between the individual setae and the surface on which the animal is moving (Russell and Johnson, 2007). This leads to an increased amount of frictional adhesive force measured on smooth surfaces relative to rough surfaces (Huber et al., 2007; Higham et al., 2019; Naylor and Higham, 2019; Cobos and Higham, 2022). Numerous studies have examined static adhesion under laboratory settings, but fewer have examined adhesive locomotion (Zaaf et al., 2001; Russell and Higham, 2009; Wang et al., 2010; Dai et al., 2011; Wang et al., 2014). Even fewer have examined locomotion of geckos in nature (Higham and Russell, 2010) and none have quantified both adhesive and locomotor performance in nature.

The genus *Rhoptropus* includes numerous diurnal species that occupy a range of rocky and sandy substrates. Their morphology reflects their ecology, in that the ground-dwelling species (*Rhoptropus afer*) is more slender, has a shorter body and longer limbs than climbing species such as *Rhoptropus bradfieldi* (Werner, 1977; Bauer et al., 1996). Indeed, the former species is able to run much faster than the latter (Higham and Russell, 2010), which is also its sister taxon. *Rhoptropus afer* also has a reduced adhesive system compared to *R. bradfieldi* (Russell and Johnson, 2013; Higham et al., 2015), reflecting its more terrestrial habits. Among species of *Rhoptropus*, *R. bradfieldi* is estimated to generate greater adhesive forces than any other species (Russell and Johnson, 2013). Combining this with the fact that the species is limited to a restricted area (Werner, 1977), often staying on a single boulder, makes them ideal for combined laboratory and field measurements.

Using *R. bradfieldi*, a relatively small diurnal basal pad-bearing gecko from the pachydactylus radiation (Haacke and Odendaal, 1981), I tested the hypothesis that adhesive performance on natural and artificial surfaces in a laboratory setting could predict escape locomotor performance in nature. I predicted that frictional adhesion on smooth acrylic is positively correlated with frictional adhesion on the natural dolerite boulder surface and that greater frictional adhesion would lead to greater maximum sprint speed and acceleration in nature. Given that there was variation in body temperature among individuals, I also examined its role in predicting speed and acceleration. Temperature has significant impacts on muscle contractile performance, which in turn can influence the speed and acceleration of lizards (Swoap et al., 1993). In light of this and previous studies of gecko performance in relation to temperature (Bergmann and Irschick, 2006), I predicted that higher body temperatures would result in higher accelerations and velocities of the climbing geckos in my study.

## MATERIALS AND METHODS

### Field site and animals

**Fig. 1.**
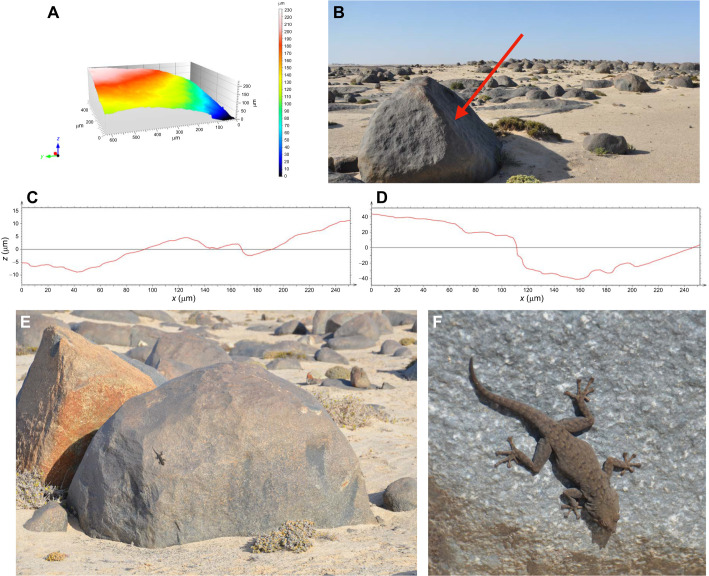
**The natural habitat of *Rhoptropus bradfieldi*.** (A) The surface of a dolerite boulder using confocal microscopy. (B) The dolerite boulder field in Namibia with the red arrow indicating a surface on which geckos were released for field escape performance. (C,D) Single transect profiles of a dolerite sample. (E) A single boulder with an individual *R. bradfieldi* in its natural position. (F) A close-up image of *R. bradfieldi*. The snout–vent length of the gecko in this image is approximately 6 cm. All photos taken by T.E.H.

I collected 13 individuals of *Rhoptropus bradfieldi* Hewitt 1935 (body mass ranging from 4.3 to 7.8 g), from a dolerite boulder field approximately 20 km north of Swakopmund along the pacific coast of Namibia (Fig. 1). Dolerite is an intrusive igneous rock, forming between existing layers of rock. When weathered, it can form boulders. In coastal Namibia, the dolerite dykes are Early Cretaceous in age (Trumbull et al., 2007). All collections were carried out with a permit from the National Commission on Research, Science and Technology (NCRST) in Namibia (permit RPIV01012019) and research was conducted using a UC Riverside IACUC animal use protocol (AUP 20200035).

Samples of the dolerite were removed from the rock and imaged using a confocal laser-scanning microscope (CLSM; LEXT OLS4000, Olympus Corporation, Japan) as in Higham et al. (2019). Samples were examined at 20× magnification and three-dimensional visualization of the surface was performed in MountainsMap Premium 7.2 (Digital Surf, France) (Fig. 1A). Multiple single line profiles were obtained (Fig. 1C,D), as was area roughness (*S*_q_).

Individual geckos were caught using a telescoping fishing rod with a slip knot at the end made from silk suture. No other diurnal geckos are found on these boulders (although *R. afer* is found on the ground around the boulders). Each gecko was immediately placed into an opaque breathable cotton bag and transported to a laboratory in Swakopmund (Ministry of Fisheries and Marine Resources). Geckos were kept less than 24 h.

### Frictional adhesion measurements

Holding the gecko by hand, the left manus of each individual was freely placed onto either acrylic or a sample of dolerite. Both were cleaned with 100% ethanol using a Kimwipe between trials. The small sample of surface was attached to a portable force gauge (MARK-10 Series M5-10, ±0.1% full-scale) and the lizard was pulled in parallel opposition until displaced. A single maximum force from five trials per individual per surface was retained for further analyses. Body mass was then measured using a portable Pesola spring scale (10 g range).

**Fig. 2.**
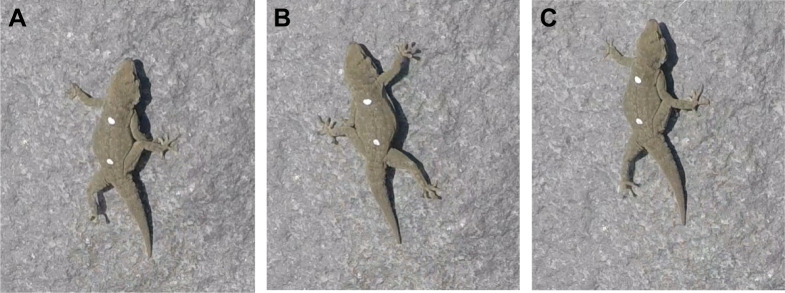
**Three sequential images of *R. bradfieldi* during an escape upon release.** (A) Footfall, (B) the end of stance and (C) the subsequent footfall images from a high-speed video sequence using a GoPro. The white dots are 18.7 mm apart. The time between A and C is approximately 100 ms.

Following the adhesive force measurements, two dots of white nail polish were painted on the dorsal surface of each gecko, approximately at the pectoral and pelvic girdles (Fig. 2). The distance between these two points was measured using calipers and was later used for calibrating the video (see below).

### Field sprint performance measurements

Each gecko was returned to the general location of capture. GoPro Hero7 cameras were aimed perpendicular to a boulder's surface and set to record at 120 frames per second. The individual was released at a field active body temperature (26 to 33°C, measured using a surface infrared thermometer) about midway between the ground and top of the boulder. The lizard was placed on the rock with its head pointing up (Fig. 2). When released, the lizards would often immediately sprint up and over the boulder. Thus, every trial involved a start from a standstill. Although the same boulder was not used among trials, I selected similarly sized boulders with comparable inclination (∼50 deg).

### Video analyses

The two points on the dorsal surface of the gecko were digitized frame-by-frame in DLTdv8a (Hedrick, 2008) in MATLAB R2023a. For each frame, the distance between the dots was measured and used as the calibration for that frame. Thus, I used a continuous calibration throughout each trial that was specific to the lizard. This proved to be more accurate than attempting to calibrate the entire field of view, given that some geckos ran up at an angle from the vertical. The relatively stout body form of these geckos meant that lateral undulation (between girdles) was minimal, so the distance between the two points was constant throughout the stride. The average per stride error (standard error divided by the mean) in the calibration (pixels mm^−1^) between the points across all individuals was 0.74% and was frequently below 0.5% and rarely above 1.0%. This could easily be attributed to digitizing error and not an actual error in the distance between points.

The displacement of the lizard between frames was calculated (using the calibration of the second frame) and then filtered using a zero-lag Butterworth filter with a cut-off frequency of 50 Hz (Penning et al., 2016; Whitford et al., 2019) using MATLAB code. From there, the instantaneous velocities and accelerations were calculated as the first and second derivative of the filtered displacement values, respectively. All raw values of speed were plotted alongside the filtered data to ensure that maximum values were similar.

The timing of footfall for the left pes was recorded in order to determine stride frequency (*f*_stride_) (1/stride duration in seconds) and stride length (*L*_stride_) (distance the anterior point travelled between subsequent footfalls). These were used to determine how speed is modulated by *R. bradfieldi*.

### Safety factor

The maximum frictional adhesive force from the right manus was multiplied by 4 in order to estimate the maximum adhesive force from the animal. Realistically, the animal would not propel itself using all four feet simultaneously, so this is an overestimate. To obtain an estimate of safety factor (SF), I first estimated locomotor force (*F*_loc_) (as in Bergmann and Irschick, 2006):
(1)

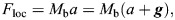
where *M*_b_ is body mass, *a* is the maximum instantaneous acceleration obtained from high-speed video in nature, ***g*** is acceleration due to gravity (9.81 m s^−2^). I then calculated the total adhesive force (*F*_adh_) by multiplying the measurement from the left manus by 4 to account for all 4 feet. SF was then calculated as:
(2)

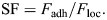


Safety factor is a key variable that likely drives the evolution of morphological traits (Higham et al., 2021a).

Finally, I calculated mass-specific power (MSP, W kg^−1^) using the following equation, as in Bergmann and Irschick (2006):
(3)

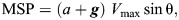
where *V*_max_ is the maximum speed of the gecko (which occurred approximately at the same time as *a*) and θ is the angle of the substrate during the escape (50 deg).

### Statistics

Multiple regressions were used to evaluate the relationships between frictional adhesive strength (independent), body temperature (independent), body mass (independent) and maximum running speed (dependent) and acceleration (dependent). Corrected AIC values were used to select the best model. Standard regression coefficients (SRCs) were extracted for all variables, as were the variance inflation factors (VIFs) to assess collinearity among variables. All statistics were run in SYSTAT 8.0. *P*<0.05 was used as the criterion for statistical significance.

## RESULTS

The area roughness (*S*_q_) of dolerite was 21.9 µm. In general, all 13 geckos all exhibited strong adhesion to acrylic, ranging from 0.4 N to 1.8 N for the left manus. On the dolerite surface, adhesion ranged from 0.04 N to 0.33 N for the left manus. Because of the relatively minimal variation in adhesion force on the dolerite surface, it was not used in the models below. However, the correlation between adhesion on the dolerite surface and the acrylic surface was significant (*R*^2^=0.25, *P*=0.047).

**Fig. 3.**
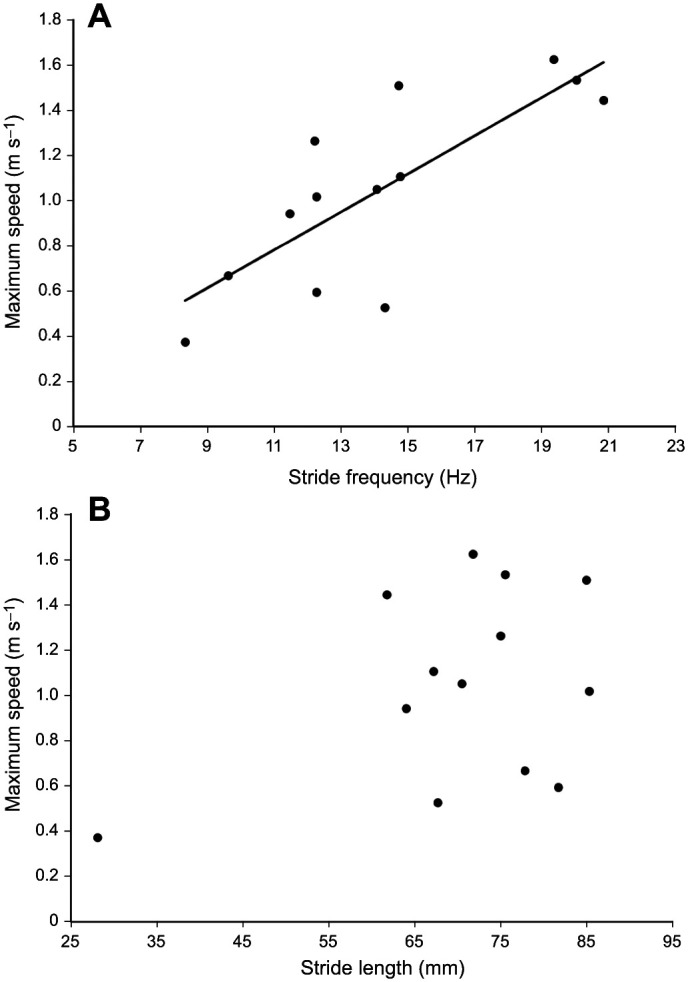
**Linear regressions of stride frequency and stride length versus maximum sprinting speed.** (A) Stride frequency and (B) stride length versus maximum sprinting speed. Stride frequency was significantly correlated with maximum speed (*R*^2^=0.61, *P*=0.002), but stride length was not (*R*^2^=0.16, *P*=0.17).

Maximum velocities ranged from 0.52 to 1.62 m s^−1^ and maximum acceleration values ranged from 5.76 to 26.98 m s^−2^. Maximum acceleration was positively correlated with maximum running speed (*R*^2^=0.50, *P*=0.007). Stride frequency was positively correlated with maximum running speed (Fig. 3A; *R*^2^=0.61, *P*=0.002), but Stride length was not (Fig. 3B; *R*^2^=0.16, *P*=0.17).

**Fig. 4.**
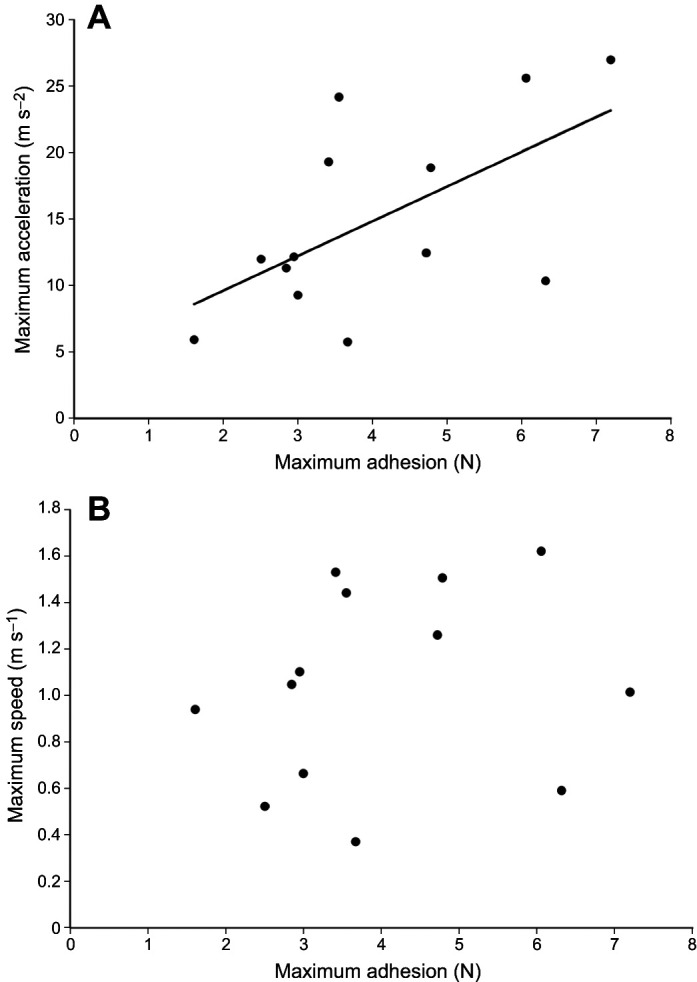
**Linear regressions of maximum adhesion on acrylic versus maximum acceleration and maximum speed.** (A) Maximum acceleration and (B) maximum speed. Adhesion was significantly correlated with acceleration (*R*^2^=0.36, *P*=0.017), but not speed (*R*^2^=0.04, *P*=0.116).

**Fig. 5.**
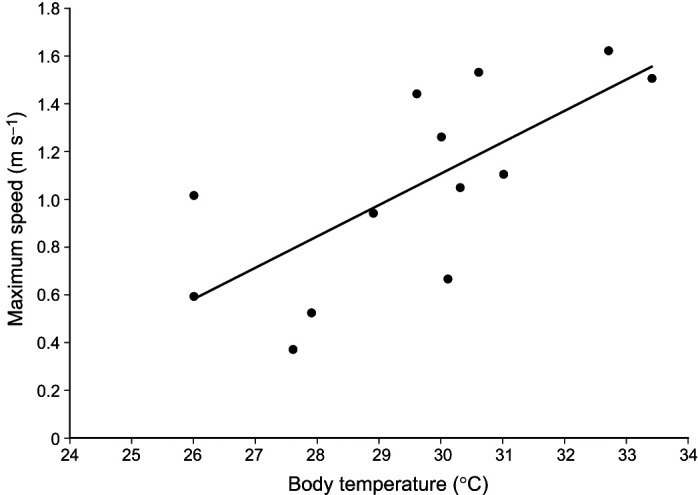
**Linear regression of body temperature versus maximum running speed in nature.** (*R*^2^=0.51, *P*=0.006).

For both multiple regression models (speed and acceleration), the power was relatively low because of the low sample size (13 individuals). There was no multicollinearity concern as the variance inflation factors were all below 1.5 for both models. Finally, all data were normally distributed, and the variance was homogeneous. The model that best predicted maximum acceleration in nature (Table 1; lowest AICc) included only adhesive strength on acrylic (Fig. 4A; *P*=0.017; SRC=0.68; VIF=1.05). Neither temperature (*P*=0.158; SRC=0.42; VIF=1.33) nor body mass (*P*=0.623; SRC=−0.14; VIF=1.28) significantly impacted maximum acceleration. The model that best predicted maximum speed in nature (Table 1) included only temperature (Fig. 5; *P*=0.006; SRC=0.82; VIF=1.33). Neither body mass (*P*=0.726; SRC=−0.08; VIF=1.28) nor adhesion (Fig. 4B; *P*=0.116; SRC=0.36; VIF=1.05) significantly impacted maximum speed.

**Table 1.** Corrected AIC values for the different multiple regression models in the study

Model	AICc (max. acceleration)	AICc (max. speed)
Adhesion+Temp+Mass	97.16	18.48
Adhesion+Temp	91.95	13.09
Adhesion	90.36*	21.14
Temp	95.36	12.38*

The model with the asterisk next to the AICc value is the best.

The safety factor while running on dolerite ranged from 0.25 to 13.02. Minor slippage occurred in individuals with lower safety factors (0.25, 0.38, 0.83). However, one individual with a low SF (0.41) did not slip and one individual with a higher SF (4.7) did slip. Mass-specific power (MSP) ranged from 4.4 W kg^−1^ to 43.9 W kg^−1^. Temperature was positively correlated, albeit marginally, with MSP (*R*^2^=0.31; *P*=0.047).

## DISCUSSION

Maximum frictional adhesive strength under optimal conditions (i.e. smooth surface) strongly predicted maximum acceleration, but not maximum speed, during escape locomotion in a day gecko from Namibia. This is the first evidence that adhesive capabilities can predict ecologically relevant performance in nature. Greater adhesive ability likely leads to a greater chance of escaping predation or capturing evasive prey, as acceleration is often more (or equally as) important than maximum speed in determining the outcome of predator–prey encounters (Webb, 1976; Elliott et al., 1977; Huey and Hertz, 1984; McElroy and McBrayer, 2010; Wilson et al., 2018). In contrast, maximum running speed was only impacted by temperature, not adhesion, highlighting that types of performance can be limited by different factors.

### Importance of acceleration

Acceleration is the ability to translate force into motion and can be limited by the mechanical power that the limb musculature can generate (Curtin et al., 2005; McElroy and McBrayer, 2010). However, this process hinges on the ability to make sufficient contact with the surface to avoid slipping. Thus, maximum acceleration (force/mass) depends on the adhesive ability of the gecko. Acceleration is key for survival where a gecko quickly dodges rapid predatory attacks by lunging terrestrial predators or aerial attacks from birds and, given the frequency of intermittent locomotion among lizards (Braña, 2003; Higham et al., 2011b), it likely plays a key general role in escape locomotion. For predator evasion, accelerating quickly is likely to be more important than maximum speed during close-range attacks. Variation in acceleration is likely driven by variation in adhesive strength, and not muscle power, in *R. bradfieldi*, given that values of safety factor frequently approached or dipped below 1. This means that the locomotor force essentially mirrors the maximum adhesive force in many cases, highlighting the limit to performance. Any increase in locomotor force would likely be met with excessive slipping and potentially falling to the ground (discussed in a subsequent section).

How does the gecko balance locomotor force so that slipping is not excessive? Given the presence of sensory receptors (sensilla) on the ventral surfaces of the toes (Bauer et al., 2023), feedback regarding the strength of contact is likely needed to avoid over-slipping during the escape. Therefore, I predict that shear-sensitive cells provide feedback about adhesive capabilities to the organism which can then determine how much propulsion to generate. For example, if frictional adhesion is strong, shear forces will be higher, and the animal may then increase acceleration within the limits of muscle power. In contrast, excessive slipping will minimize the force generated and keep the gecko moving forward, albeit at a lower acceleration. Although slipping occurred in a few of the geckos in this study, it was never excessive.

Aspects of the habitat are known to impact maximum acceleration. For example, *Anolis* lizards exhibit a reduction in maximum acceleration as perches become narrower (Vanhooydonck et al., 2006). In a study of six lacertid lizards, the texture of the substrate significantly influences acceleration capacity whereby species accelerated slower on sandy surfaces compared with cork and slate (Vanhooydonck et al., 2015). This general influence of substrate properties on acceleration does not appear universal, as surfaces of different compliance do not impact level acceleration in *Uma scoparia* and *Callisaurus draconoides* (Korff and McHenry, 2011). Additionally, incline has no impact on maximum acceleration in *C. draconoides* (Irschick and Jayne, 1998) and is minimal for the agamid *Stellio stellio* (Huey and Hertz, 1984).

Research connecting surface properties and acceleration in geckos is lacking. In a study of gecko acceleration ability on substrates of different textures, Vanhooydonck et al. (2005) found that geckos accelerate faster on smoother surfaces where greater contact between the adhesive system and surface is possible (Vanhooydonck et al., 2005). The current study adds to this in that those geckos that can adhere more strongly to a given surface will also accelerate faster. Thus, there is a complex interplay between surface structure and adhesive ability when it comes to ecological performance. Future work should continue to explore these relationships across a range of species and substrate characteristics in both the lab and field.

### Modulation of running speed

Lizards run at different maximum speeds, and these speeds are determined by differences in *f*_stride_, *L*_stride_, or a combination of the two. Several factors, such as maximum running speed or microhabitat use, can influence whether *f*_stride_ or *L*_stride_ rises to increase speed in lizards. The limit to *f*_stride_ is often attributed to the properties of skeletal muscles involved in propulsion, such as the force/velocity relationships and the maximal activation of muscles. The contact time is reduced with higher values of *f*_stride_, increasing the peak force for a given speed. It is thought that lizards with higher maximum running speeds will likely modulate *L*_stride_ rather than (or after) *f*_stride_. For example, subspecies of the Spanish wall lizard (*Podarcis hispanica*) that occupy islands or the mainland differ in how they increase speed. Island populations utilize changes in *f*_stride_, whereas mainland populations alter *L*_stride_ (Van Damme et al., 1998). Because the mainland populations achieved higher maximum speeds, it was thought that the modulation strategies depended upon predator pressure (higher on the mainland). The fastest lizards in that study achieved running speeds of approximately 30 body lengths s^−1^ by increasing *L*_stride_ (Van Damme et al., 1998). The authors highlight that *f*_stride_ modulators likely reach their maximum frequency at lower speeds than *L*_stride_ modulators. However, the geckos in my study, which modulated speed via *f*_stride_ and not *L*_stride_, achieved speeds of approximately 35 body lengths s^−1^. Thus, *R. bradfieldi* reaches similar (if not higher) speeds than *P. hispanica* despite relying on the modulation of *f*_stride_ rather than *L*_stride_. Considering this, future work should examine differences in skeletal muscle physiology between day geckos and other lizards. I predict that *R. bradfieldi* may contain muscles capable of contracting faster, perhaps because of an increase in the proportion (and diameter) of fast glycolytic muscle fibers within stance phase muscles (Higham et al., 2011a).

The microhabitat in which a lizard lives can also impact how speed is modulated. For example, among 11 species of lacertid lizards, those occupying open habitats modulate speed through changes in *L*_stride_, whereas those occurring in patches of vegetation (more cluttered) mainly increase *f*_stride_ to sprint faster (Vanhooydonck et al., 2002). The species that modulate *f*_stride_ likely need to change direction fast and frequently, and this can only be done when the feet are in contact with the ground (Vanhooydonck et al., 2002). Given the similarity of these species to *R. bradfieldi* in the current study, I conclude that dolerite boulders should be categorized as more cluttered than running on the ground in more open spaces. The three-dimensionality of a boulder is such that frequent changes in direction are likely needed since the field of view is perpetually limited.

### Thermal dependence of escape performance

Temperature is known to impact the contractile dynamics of skeletal muscle and running speed of lizards (Bennet, 1984; Marsh and Bennett, 1985, 1986; Swoap et al., 1993). The thermal dependence of muscle contraction stems from the fact that myosin ATPase activity and Ca^2+^ sequestration by the sarcoplasmic reticulum are both highly temperature dependent (Rall and Woledge, 1990). *f*_stride_ depends on contraction velocity, and *f*_stride_ was the only determinant of maximum running speed in the current study. Coincidentally, temperature also impacted maximum speed and not acceleration. Overall, it appears that maximum acceleration, in the case of pad-bearing geckos, is likely to be limited by adhesive force, not muscle contractile dynamics, when moving on sub-optimal surfaces when values of safety factor are low.

Interestingly, my results are not similar to those for *Phelsuma dubia* running on a vertical smooth surface under different temperatures (Bergmann and Irschick, 2006). Whereas these authors found that maximum acceleration was significantly increased as temperature increased, I found no such relationships. What might explain the differences between studies? Although *Phelsuma* and *Rhoptropus* are relatively closely related in the gekkotan phylogeny (Gamble et al., 2012) and they share a lack of functional claws, the conditions in which they were running differed between the studies. Future work that examined locomotion of *Phelsuma* under natural conditions, or *Rhoptropus* in laboratory conditions, will potentially reveal similar results.

Like Bergmann and Irschick (2006), I found a significant correlation between MSP and temperature, such that increases in temperature resulted in greater MSP. However, the relationship was less pronounced, perhaps due to the lack of correlation between temperature and acceleration (a component of the MSP equation). The fact that MSP rose with temperature is not necessarily surprising. The power output optimum for fast glycolytic fibers of the iliofibularis muscle in *Dipsosaurus dorsalis* was at the higher end of the temperature range (40–42°C) (Marsh and Bennett, 1985; Swoap et al., 1993).

### Combining habitat variables and adhesion

In the real world, geckos are interacting with substrates that vary considerably in roughness, and what determines the underlying roughness might also vary among substrates (Higham et al., 2019). Because species of the genus *Rhoptropus* lack functional claws, my study reveals the impact of the adhesive system alone. This is in contrast to geckos that have functional claws, in which minimal adhesive performance could occur on a surface with intermediate roughness due to the lack of adhesive or claw contact (Naylor and Higham, 2019; Pillai et al., 2020a; Pamfilie et al., 2023). In other words, adhesion could dominate clinging on smooth surfaces, whereas claws dominate on very rough surfaces.

Slipping during an escape might also depend upon the roughness encountered. As noted above, slipping did occur in some individuals, including three of the five individuals with an SF below 1. The two with the lowest values of SF appeared to slip the most. Interestingly, two lizards with an SF below 1 did not exhibit slipping, and an individual with a relatively high SF (4.7) did exhibit slipping. What could drive this variation? It is not possible to control the path that geckos take when videorecording them escaping in nature. Perhaps these lizards encountered different degrees of roughness during their escape, as rocks are rarely uniform in roughness at small scales. This inherent variability is one of the drawbacks of quantifying only locomotion in nature. Matching this with laboratory studies that control roughness and keep it uniform, could tease apart the impact of roughness on locomotion. In nature, one might observe the escape trajectory and then using a molding process to quantify the roughness experienced. One could then obtain values of roughness alongside the escape path, but not used by the lizard. This would reveal whether geckos select escape paths that minimize roughness. This would, of course, rely on the ability of the geckos to ‘map’ their habitat and establish optimal escape routes. Recent work has shown that lizards do have spatial memory (LaDage et al., 2012), but it is not clear how much resolution *R. bradfieldi* might have.

Within *Rhoptropus afer*, a closely related species to *R. bradfieldi*, recent work found that the morphology of the adhesive system varies in relation to the microhabitat of the population. Those individuals that encounter more horizontal surfaces exhibited the greatest reduction of the adhesive system (Collins et al., 2015). Thus, phenotypic plasticity may play a role in the relationships between adhesive and locomotor performance in *R. bradfieldi*. The current study examined geckos within a single area, but this species occupies different types of rocks at other locations. Future work could determine if habitat drives adhesive performance, which then impacts locomotor performance.

How might clinging performance be linked to habitat use and performance more generally? Recent research with *Anolis cristatellus* found that hindlimb toepad area was positively correlated with running performance on a wood surface with gradual inclination (Winchell et al., 2018). Thus, it appears that, at least in some cases, adhesive performance (based on toepad area) can be used to predict performance. Another recent study using three species of *Oedura* geckos found that clinging performance in the arboreal and saxicolous species was greater on coarse than on fine sandpaper, and they selected microhabitats in the laboratory on which their clinging performance was high (Pillai et al., 2020b). Toepad area is not a good predictor of adhesion when comparing across disparate groups of lizards given the potential for different morphological configurations within the toepads. For example, anoles tend to have thinner, shorter and more densely packed setae that terminate in a single spatulate tip (Garner et al., 2021). Geckos, on the other hand, exhibit hierarchically branched setae with hundreds of spatulate tips. This has significant impact on the relationship between toepad area and adhesive performance. In a recent study that included both a gecko (*Phelsuma laticauda*) and anoles (*Anolis carolinensis* and *Anolis sagrei*), toepad area was smaller in the gecko but its adhesive performance was the highest (Wright et al., 2021). Thus, clinging performance should be used when comparing species from different genera.

Wright et al. (2021) also examined the relationship between clinging performance and habitat use in geckos and anoles. Of the one species of gecko and two species of anole, *A. sagrei* exhibited the greatest clinging performance on rough substrates (likely due to the presence of claws) and used rough perches almost exclusively in semi-natural enclosures in Hawaii. In contrast, *P. laticauda* adhered best on smooth surfaces and they were observed on smooth surfaces 75% of the time (Wright et al., 2021). Given that gecko species vary considerably in their ability to adhere (Irschick et al., 1996; Higham et al., 2017), future work could examine the relationship between habitat use and adhesive ability.

### Limitations to the study

Several limitations to this study could and should be addressed in future research. First, the power of the models was a bit low, which is due to the sample size of 13. Future work should expand on this sample size. Another limitation is the inability to know exactly what roughness each gecko experienced, which could in turn influence their escape performance. Future work could evaluate the roughness of the escape trajectory (using molding techniques in the field) and then add this as a variable to future models. Finally, the frame rate of 120 frames s^−1^ could be increased to 240 or even 500 in future studies. Although the frame rate used in this study might impact the magnitude of acceleration, the correlations with other variables should not be impacted.

### Conclusions and future directions

I showed that different measures of performance are limited by different factors. Maximum acceleration is limited only by maximum adhesive capability, whereas maximum speed is limited by stride frequency, which is ultimately influenced by temperature. Acceleration, which appears temperature-insensitive, is potentially key for escaping predation. This means that geckos will be equally successful at escaping predation across a range of ambient environmental temperatures, but the ability to adhere is likely to influence fitness. This latter point remains to be tested but will be an important next step.

The data presented here are for a single species at a single location in the habitat. Will these results be comparable to other species in the genus that live on different surfaces? Members of the genus *Rhoptropus* all lack functional claws and occupy a range of substrates across Namibia and Angola, including sedimentary rock (sandstone, conglomerate and dolomite), metamorphic rocks (schist and gneiss), igneous rocks (granite, diorite, and gabbro), and even loose sand on the ground (Werner, 1977; Odendaal, 1979; Haacke and Odendaal, 1981; Bauer and Good, 1996; Bauer et al., 1996; Russell and Johnson, 2013; Collins et al., 2015). Thus, they would thus be an excellent group for exploring such questions, including how rock type and surface properties influence the relationship between adhesion and performance.

## Supplementary Material

10.1242/jeb.247906_sup1 Supplementary information
