# Supplementary material for: Frictional adhesion of geckos predicts maximum running performance in nature
Source: J Exp Biol. 2025 Jan 9;228(1):jeb247906. doi: 10.1242/jeb.247906 (PMC11744320; doi:10.1242/jeb.247906)
Supplement: Supplementary information [file jexbio-228-247906-s1.pdf]

Table S1. Individual data from this study.

Individual	Maximum velocity m/s	Max acceleration m/s/s	Body temperature Degrees Celsius	Adhesion on Acrylic Newtons	Body mass Grams
1	1.10	12.15	31.0	2.94	7.15
2	1.62	25.59	32.7	6.05	7.85
3	0.37	5.76	27.6	3.66	6.35
4	0.94	5.93	28.9	1.61	7.35
5	0.67	9.28	30.1	2.99	5.55
7	1.44	24.16	29.6	3.54	4.45
8	1.05	11.30	30.3	2.84	5.15
9	1.02	26.98	26.0	7.18	6.00
10	0.59	10.34	26.0	6.31	4.30
11	0.52	12.00	27.9	2.50	4.50
12	1.51	18.86	33.4	4.78	6.40
13	1.26	12.44	30.0	4.71	5.40
14	1.53	19.30	30.6	3.41	5.50

Journal of Experimental Biology • Supplementary information
